# Supplementary material for: G protein-coupled receptor 151 regulates glucose metabolism and hepatic gluconeogenesis
Source: Nat Commun. 2022 Dec 1;13:7408. doi: 10.1038/s41467-022-35069-9 (PMC9715671; doi:10.1038/s41467-022-35069-9)
Supplement: Supplementary file 1 — Supplementary Information [file 41467_2022_35069_MOESM1_ESM.pdf]

G protein-coupled receptor 151 regulates glucose metabolism and hepatic gluconeogenesis

Ewa Bielczyk-Maczynska[1,2,3,*], Meng Zhao[2,3,4], Peter-James H. Zushin[5], Theresia M. Schnurr[1,2,3], Hyun-Jung Kim[1], Jiehan Li[1,2,3], Pratima Nallagatla[6], Panjamaporn Sangwung[1,2,3], Chong Park[1,2,3], Cameron Cornn[1], Andreas Stahl[5], Katrin J. Svensson[2,3,4], and Joshua W. Knowles[1,2,3,7,*]

1. Division of Cardiovascular Medicine, Department of Medicine, Stanford University School of Medicine, Stanford, CA, USA.
2. Stanford Diabetes Research Center, Stanford University School of Medicine, Stanford, CA, USA.
3. Stanford Cardiovascular Institute, Stanford University School of Medicine, CA, USA.
4. Department of Pathology, Stanford University School of Medicine, Stanford, CA, USA.
5. Department of Nutritional Sciences and Toxicology, University of California at Berkeley, Berkeley, CA, USA.
6. Genetics Bioinformatics Service Center, Stanford University School of Medicine, Stanford, CA, USA.
7. Stanford Prevention Research Center, Stanford University School of Medicine, Stanford, CA, USA.

* Corresponding authors: Ewa Bielczyk-Maczynska (ewabm@stanford.edu), Joshua W. Knowles (knowlej@stanford.edu).

Supplementary Information

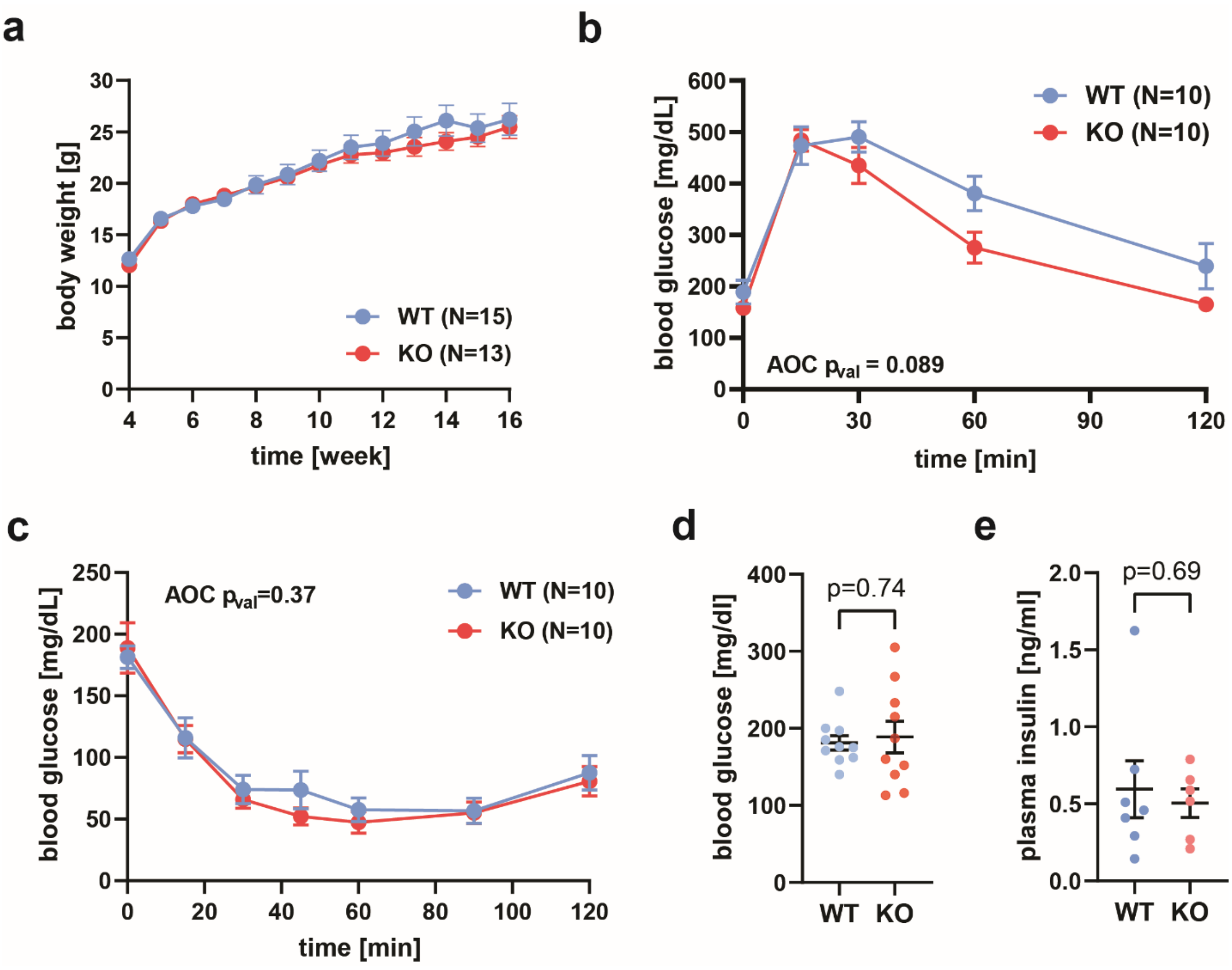

Extended Data Fig. 1 | Supplementary data corresponding to Fig. 1 for DIO females. a, Body weight in female DIO KO and WT mice over 12 weeks of HFD (N=15, WT; N=13, KO). **b**, Blood glucose levels measured during glucose tolerance testing in *Gpr151* KO and WT DIO females. Area of the curve (AOC) compared using two-tailed Student *t* test (N=10, WT; N=10, KO). **c**, Blood glucose levels measured during insulin tolerance testing in *Gpr151* KO and WT in DIO female mice. AOC compared using two-tailed Student *t* test (N=10, WT; N=10, KO). **d**, Fasting glucose levels in *Gpr151* WT and KO DIO female mice measured in whole blood (N=10, WT; N=10, KO). Two-tailed Student *t* test. **e**, Fasting insulin levels measured in blood plasma of DIO female mice (N=7, WT; N=6, KO). Two-tailed Student *t* test. **a-e**, All data are presented as mean values +/- SEM. Source data are provided as a Source Data file.

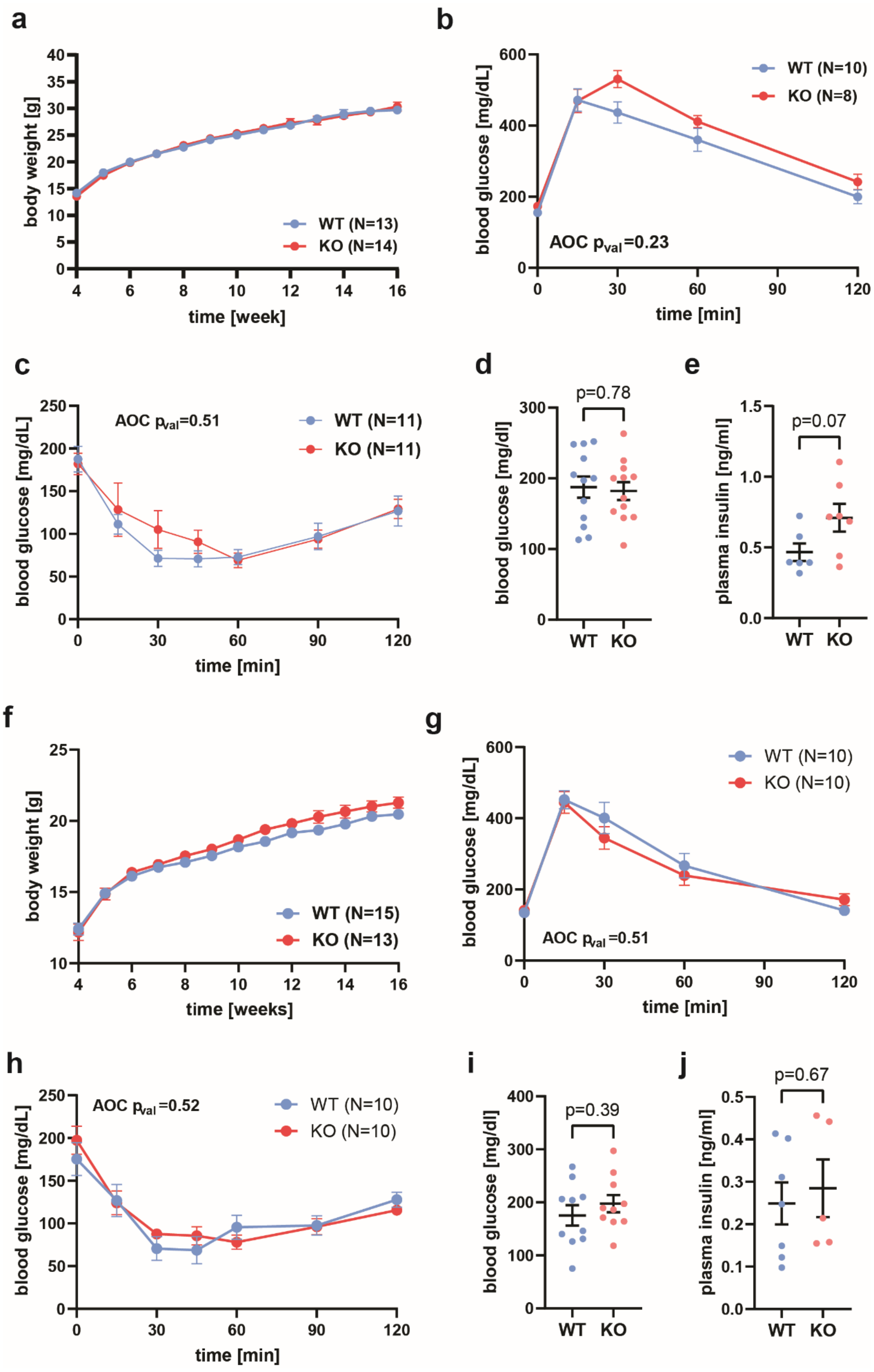

a
body weight [g]
time [week]
WT (N=13)
KO (N=14)
b
blood glucose [mg/dL]
time [min]
WT (N=10)
KO (N=8)
AOC p_{val}=0.23
c
blood glucose [mg/dL]
time [min]
AOC p_{val}=0.51
WT (N=11)
KO (N=11)
d
blood glucose [mg/dl]
p=0.78
WT
KO
e
plasma insulin [ng/ml]
p=0.07
WT
KO
f
body weight [g]
time [weeks]
WT (N=15)
KO (N=13)
g
blood glucose [mg/dL]
time [min]
WT (N=10)
KO (N=10)
AOC p_{val}=0.51
h
blood glucose [mg/dL]
time [min]
AOC p_{val}=0.52
WT (N=10)
KO (N=10)
i
blood glucose [mg/dl]
p=0.39
WT
KO
j
plasma insulin [ng/ml]
p=0.67
WT
KO

Extended Data Fig. 2 | Supplementary data corresponding for Fig. 1 for SD-fed mice. a-e, SD-fed males. **a**, Body weight in male KO and WT mice over 12 weeks of SD. **b**, Blood glucose levels measured during glucose tolerance testing in *Gpr151* KO and WT SD males. Area of the curve (AOC) compared using two-tailed Student *t* test. **c**, Blood glucose levels measured during insulin tolerance testing in *Gpr151* KO and WT in SD male mice. AOC compared using two-tailed Student *t* test. **d**, Fasting glucose levels in *Gpr151* WT and KO DIO SD-fed male mice measured in whole blood (N=12, WT; N=12, KO). Two-tailed Student *t* test. **e**, Fasting insulin levels measured in blood plasma of SD male mice (N=6, WT; N=7, KO). Two-tailed Student *t* test. **f-j**, SD-fed females. **f**, Body weight in female KO and WT mice over 12 weeks of SD. **g**, Blood glucose levels measured during glucose tolerance testing in *Gpr151* KO and WT SD females. Area of the curve (AOC) compared using two-tailed Student *t* test. **h**, Blood glucose levels measured during insulin tolerance testing in *Gpr151* KO and WT in SD female mice. AOC compared using two-tailed Student *t* test. **i**, Fasting glucose levels in *Gpr151* WT and KO DIO SD-fed female mice measured in whole blood (N=10, WT; N=10, KO). Two-tailed Student *t* test. **j**, Fasting insulin levels measured in blood plasma of SD female mice (N=7, WT; N=5, KO). Two-tailed Student *t* test. **a-j**, All data are presented as mean values +/- SEM. Source data are provided as a Source Data file.

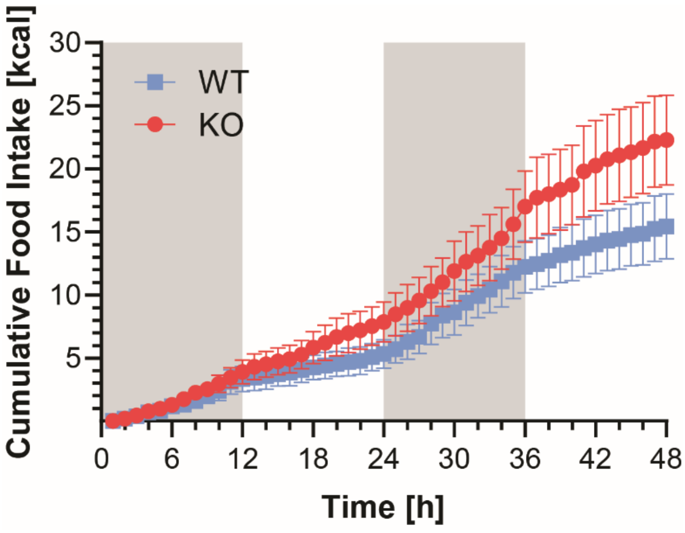

Extended Data Fig. 3 | Increased food intake in *Gpr151* KO mice compared to WT littermates in the male DIO cohort of mice assessed using CLAMS. Representative graph of cumulative food intake over 48 h of measurement (N=5, WT; N=4, KO). Data are presented as mean values +/- SD. Source data are provided as a Source Data file.

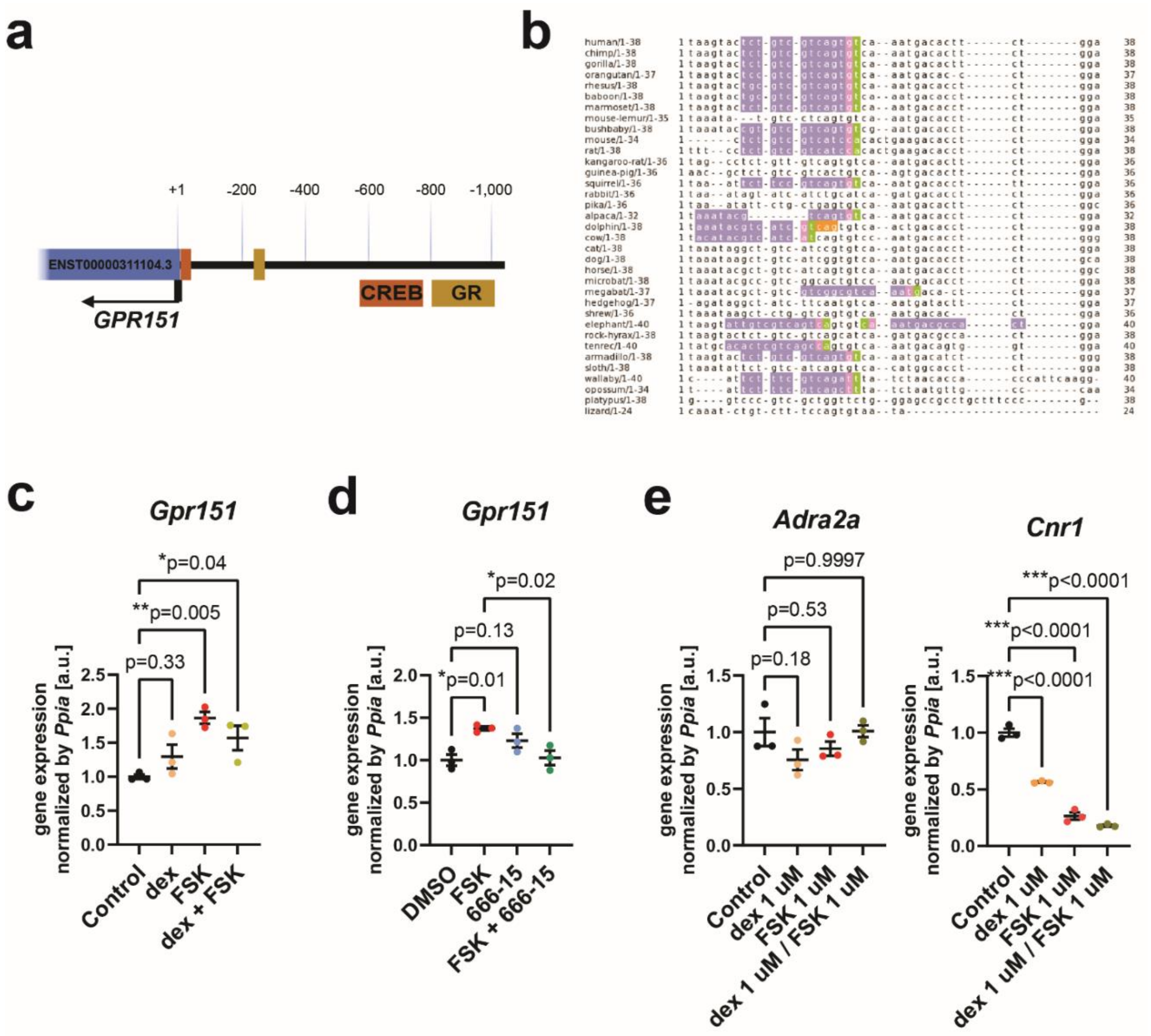

Extended Data Fig. 4 | *Gpr151* expression is regulated by cAMP through CREB. a, Location of the conserved CREB and GR motifs in the proximal promoter of the human *GPR151* gene. The sites conserved between mouse and human genes are shown. **b**, Analysis of promoter sequence of *GPR151*, conducted using Contra v3 for 1kb upstream of the human transcript. The alignment of conserved sequences 1-38 bp downstream of TSS. CREB binding sites are highlighted. **c**, RT-qPCR quantification of *Gpr151* expression in AML12 cells following 2 h treatment with 1 μM dexamethasone (dex), 1 μM forskolin (FSK), or both. Data from one experiment representative for two independent experiments. n=3 technical replicates. Ordinary one-way ANOVA with Dunnett's multiple comparisons test (p-value=0.0099). **d**, RT-qPCR quantification of *Gpr151* expression following 3 h treatment with 10 μM FSK, 500 nM CREB inhibitor 666-15, or both. Data from one experiment representative for three independent experiments. n=3 technical replicates. Ordinary one-way ANOVA with Sidak's multiple comparisons test (p-value=0.014). **e,** RT-qPCR quantification of the expression of *Adra2a* and *Cnr1* in response to 2 h treatment with dexamethasone, forskolin, or both. The expression of *Adra2b* and *Adra2c* was not detected. Data from one experiment representative for two independent experiments. n=3 technical replicates. Ordinary one-way ANOVA with Sidak's multiple comparisons test (*Adra2a* p-value=0.2 ; *Cnr1* p-value<0.0001). **c-e**, All data are presented as mean values +/- SEM. Source data are provided as a Source Data file.

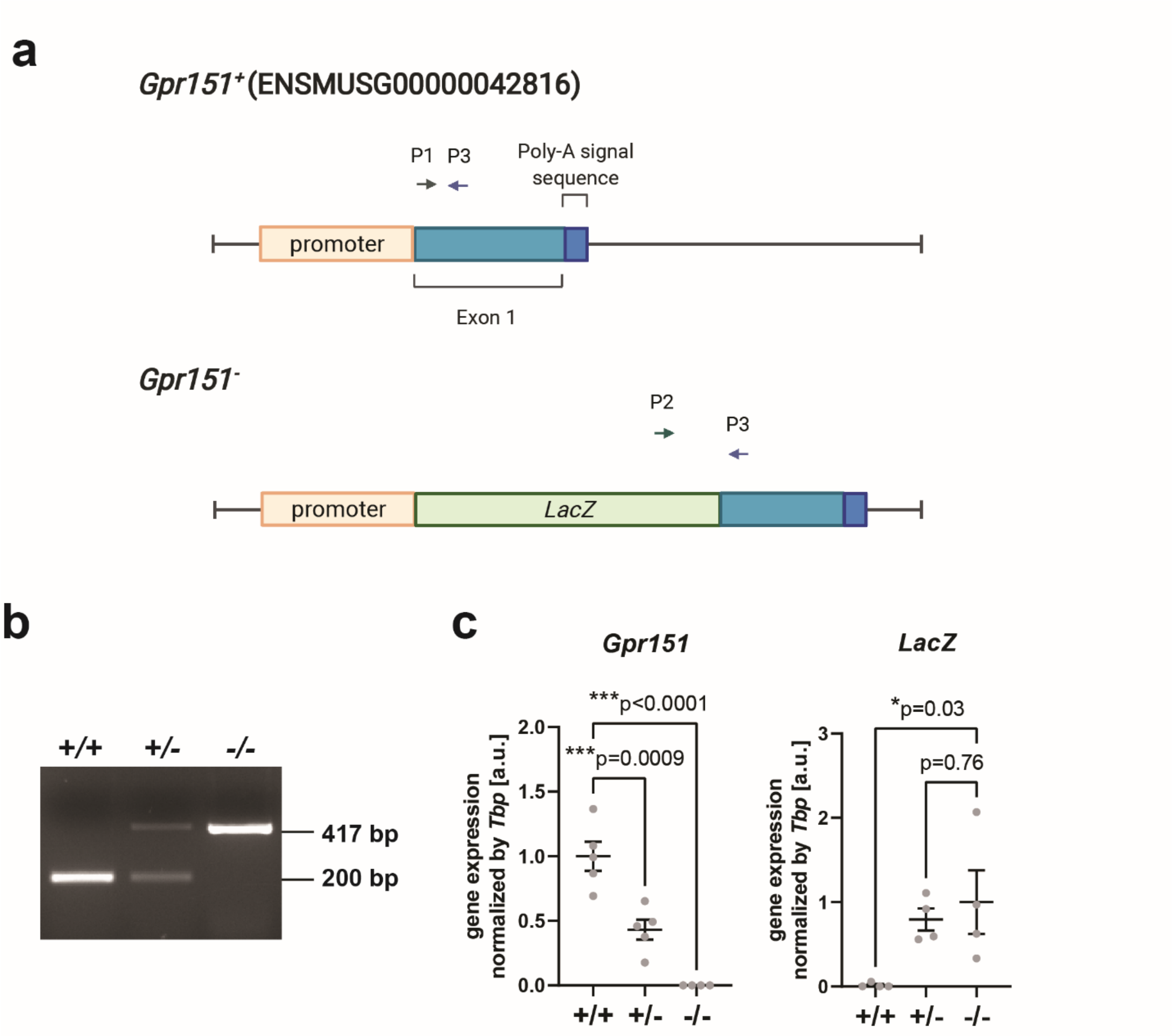

Extended Data Fig. 5 | Analysis of the *Gpr151* KO allele in the liver. a, Schematic of the wild-type (*Gpr151*+) and knockout (*Gpr151*-) alleles of the *Gpr151* gene. Location of the primers used for genotyping (two forward primers P1 and P2 and a common reverse primer P3) is indicated. **b**, Representative image of agarose gel electrophoresis of the PCR genotyping product. Sanger sequencing of the PCR product for *Gpr151* KO (-/-) confirms the disruption of the gene. Data from one experiment representative for three independent experiments. **c**, RT-qPCR quantification of *Gpr151* and *LacZ* expression in the livers of 16-week-old SD-fed male mice. Data normalized to the gene expression in WT and KO mice, respectively (N=4, WT; N=5, HET; N=5, KO). One-way ANOVA with Dunnett's multiple comparisons test (*Gpr151* p-value<0.0001; *LacZ* p-value=0.0342). Data are presented as mean values +/- SEM. Source data are provided as a Source Data file.

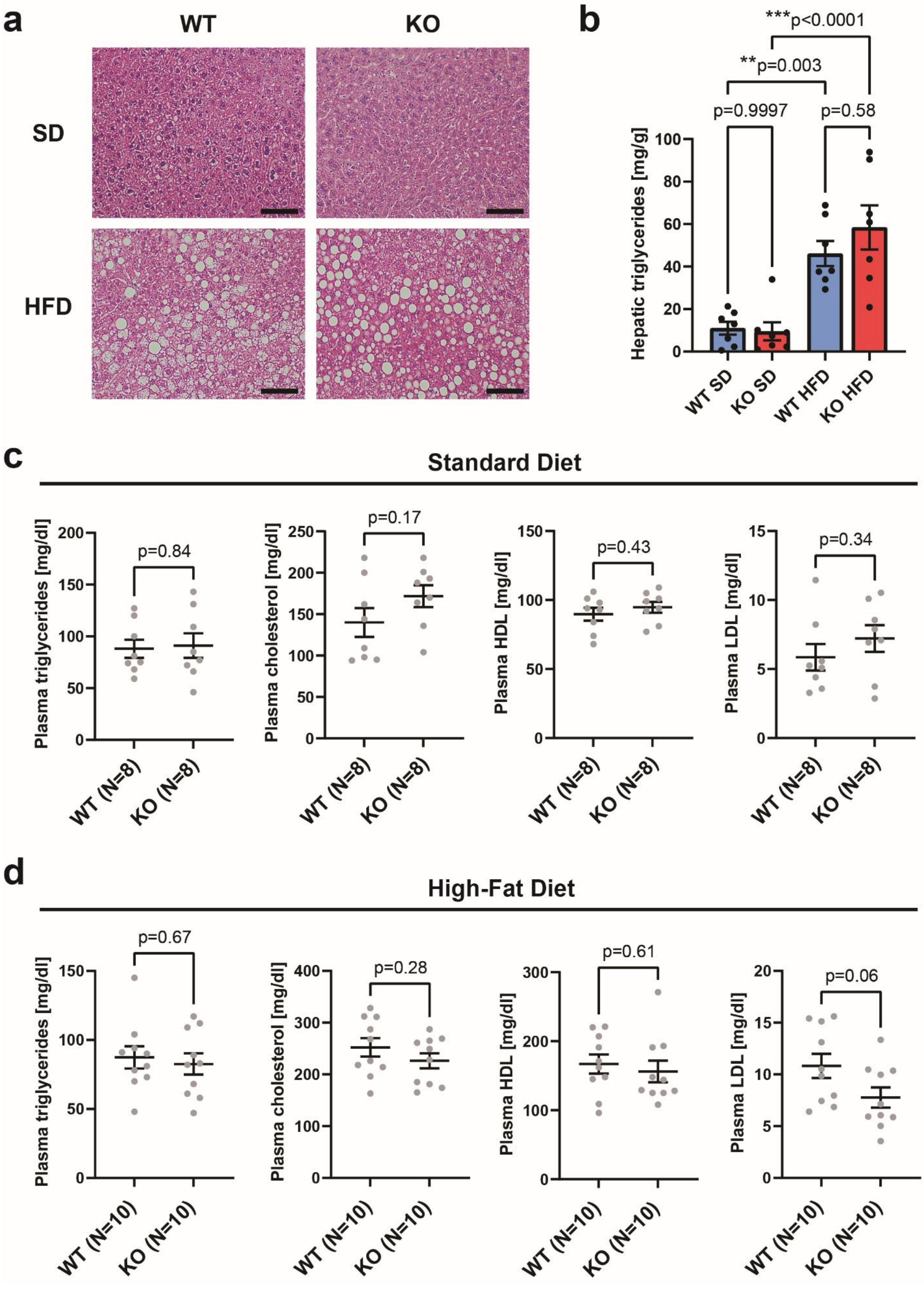

a
WT
KO
SD
HFD
b
***p<0.0001
**p=0.003
p=0.9997
p=0.58
Hepatic triglycerides [mg/g]
0
20
40
60
80
100
WT SD
KO SD
WT HFD
KO HFD
c
Standard Diet
Plasma triglycerides [mg/dl]
p=0.84
Plasma cholesterol [mg/dl]
p=0.17
Plasma HDL [mg/dl]
p=0.43
Plasma LDL [mg/dl]
p=0.34
WT (N=8)
KO (N=8)
d
High-Fat Diet
Plasma triglycerides [mg/dl]
p=0.67
Plasma cholesterol [mg/dl]
p=0.28
Plasma HDL [mg/dl]
p=0.61
Plasma LDL [mg/dl]
p=0.06
WT (N=10)
KO (N=10)

Extended Data Fig. 6 | Analysis of liver morphology and lipid metabolism in *Gpr151* KO mice. **a**, Representative images of H&E staining of livers from SD- and HFD-fed 16-week-old male mice. Scale: 250 µm. Images representative for N=8, WT; N=8, KO. **b**, Quantification of liver triglycerides in SD- and HFD-fed 16-week-old male mice (N=7, WT; N=7, KO). Ordinary one-way ANOVA with Sidak's multiple comparisons test (p-value<0.0001). **c**, Quantification of total triglycerides, total cholesterol, HDL and LDL in the plasma of SD- fed male mice at 16 weeks of age. Two-tailed Student *t* tests. **d**, Quantification of total triglycerides, total cholesterol, HDL and LDL in the plasma of HFD- fed male mice at 16 weeks of age. Two-tailed Student *t* tests. The number of mice is indicated. **b-d**, All data are presented as mean values +/- SEM. Source data are provided as a Source Data file.

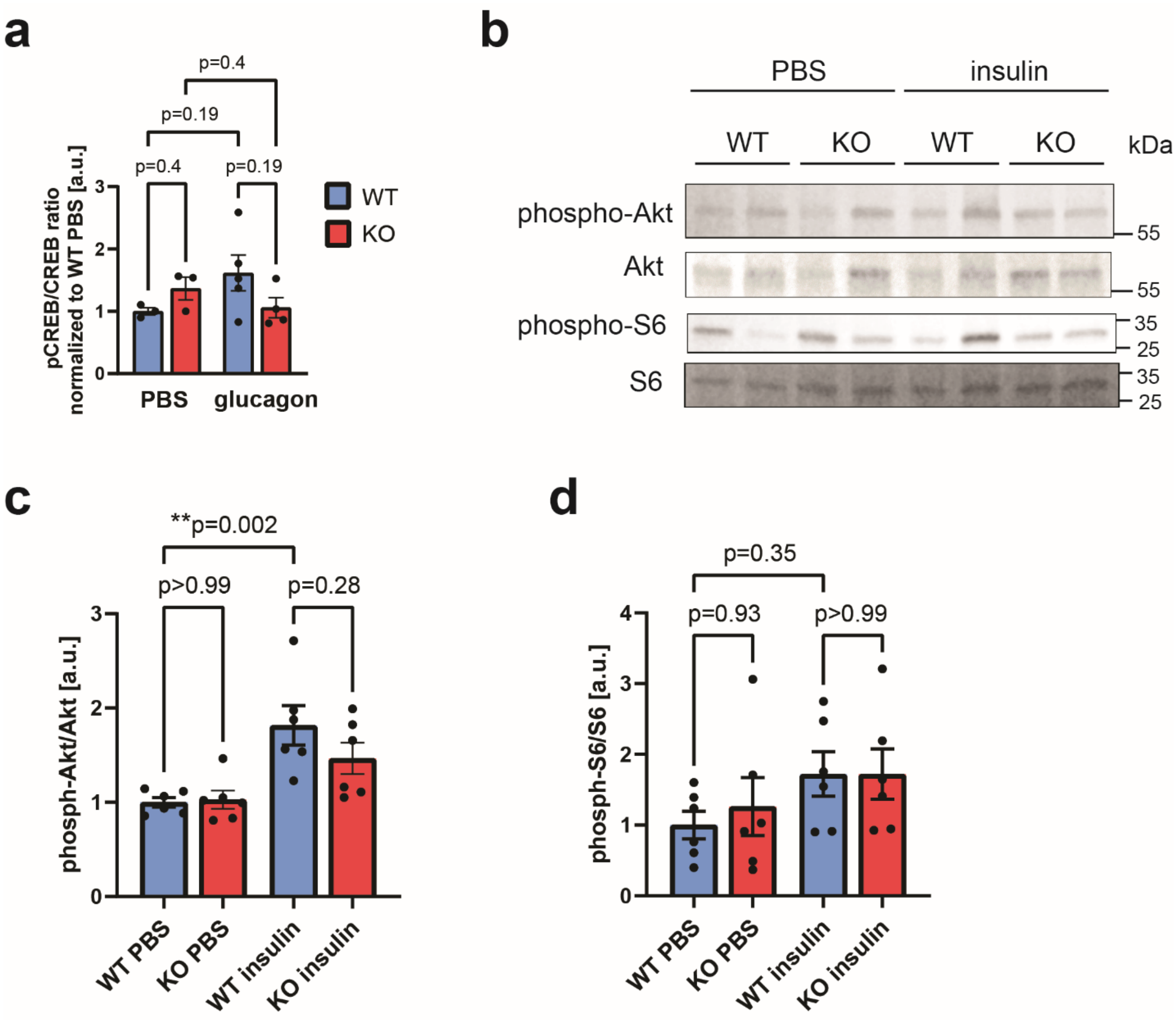

Extended Data Fig. 7 | Downstream signaling from glucagon and insulin receptors in the livers of *Gpr151* WT and KO mice. a, Quantification of the ratio between phosphorylated CREB (Ser133) and total CREB, normalized to PBS-injected WT mice, in the livers of PBS- and glucagon-injected mice (N=3, WT PBS; N=3, KO PBS; N=5, WT glucagon; N=4, KO glucagon). Ordinary one-way ANOVA with Sidak's multiple comparisons test (p-value=0.23). Data are presented as mean values +/- SEM. **b**, Western blotting of phosphorylated S6, total S6, phosphorylated Akt and total Akt in the total protein fraction isolated from the livers of PBS- and insulin-injected *Gpr151* WT and KO eight-week-old male mice. Samples were run on the same blot. Data from one experiment representative for three independent experiments. **c**, Quantification of the phosphorylated to total Akt ratio in the livers of PBS- and insulin-injected mice (N=6, WT PBS; N=6, KO PBS; N=6, WT insulin; N=6, KO insulin). Ordinary one-way ANOVA with Sidak's multiple comparisons test (p-value=0.0018). Data are presented as mean values +/- SEM. **d**, Quantification of the phosphorylated to total S6 in the livers of PBS- and insulin-injected mice (N=6, WT PBS; N=6, KO PBS; N=6, WT insulin; N=6, KO insulin). Ordinary one-way ANOVA with Sidak's multiple comparisons test (p-value=0.34). Data are presented as mean values +/- SEM. Source data are provided as a Source Data file.

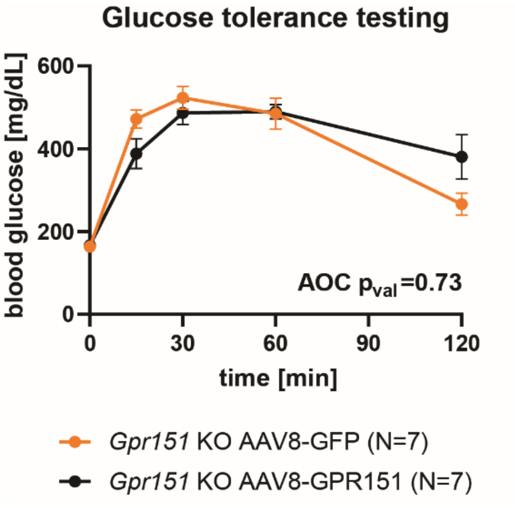

Extended Data Fig. 8 | Supplementary data to Figure 6. Blood glucose levels of *Gpr151* KO mice injected with AAV8-GPR151 and AAV8-GFP subjected to glucose tolerance testing (N=7, *Gpr151* KO AAV8:GFP; N=7, *Gpr151* KO AAV8:GPR151). AOC compared using two-tailed Student *t* test. Data are presented as mean values +/- SEM. Source data are provided as a Source Data file.

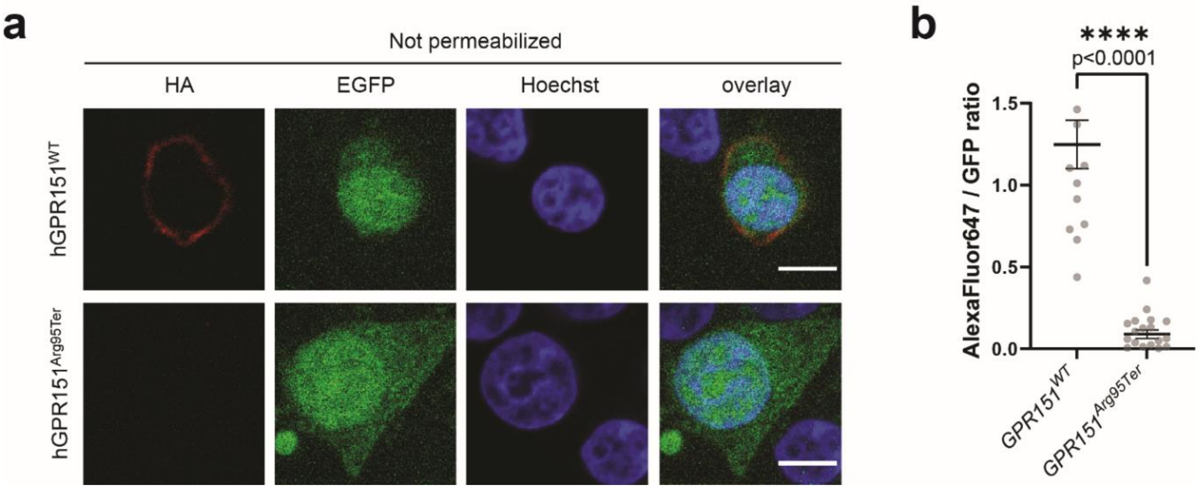

Extended Data Fig. 9 | Supplementary Data to Figure 7. **a**, Confocal images of EGFP-positive cells that were transfected with each of the plasmids, fixed and fluorescently stained against the HA tag without permeabilization. Data representative for three independent experiments. Nuclei counterstained with Hoechst. Images from a single z-plane, 60x objective. Scale bar: 10 um. **b**, Quantification of the ratio between total HA staining (AlexaFluor647) and total GFP signal over the entire cell area in a single confocal z-plane (n=14, GPR151^{WT}; n=20, GPR151^{Arg95Ter}). Data are presented as mean values +/- SEM. Two-tailed Student *t* test; ****, p<0.0001. Source data are provided as a Source Data file.

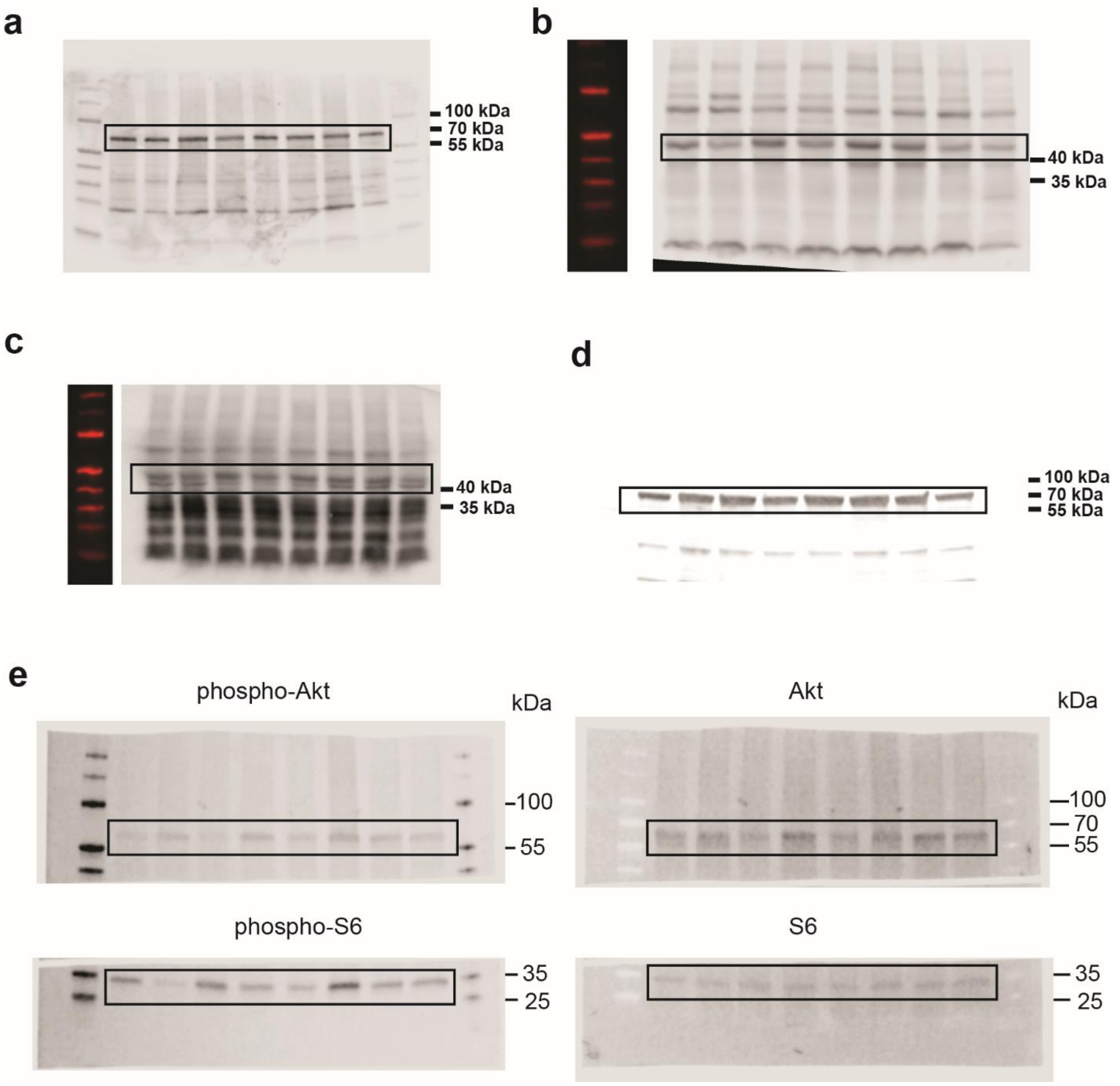

Extended Data Fig. 10 | Images of uncropped blots. a, Corresponding to Figure 3h. **b**, Corresponding to Figure 5a (p-CREB). **c**, Corresponding to Figure 5a (CREB). **d**, Corresponding to Figure 6e. **e**, Corresponding to Extended Data Figure 7b. Boxed areas indicate the cropped image used in the figures. PageRuler Prestained Protein Ladder, 10 to 180 kDa (Thermo Fisher Scientific) used. In red: ladder in the same blot visualized in the 700 nm channel for size comparison.
