# Supplementary material for: G protein-coupled receptor 151 regulates glucose metabolism and hepatic gluconeogenesis
Source: Nat Commun. 2022 Dec 1;13:7408. doi: 10.1038/s41467-022-35069-9 (PMC9715671; doi:10.1038/s41467-022-35069-9)
Supplement: Supplementary file 2 — Reporting Summary [file 41467_2022_35069_MOESM2_ESM.pdf]

nature portfolio

Corresponding author(s): Knowles, Joshua; Bielczyk-Maczynska, Ewa

Last updated by author(s): Oct 14, 2022

Reporting Summary

Nature Portfolio wishes to improve the reproducibility of the work that we publish. This form provides structure for consistency and transparency in reporting. For further information on Nature Portfolio policies, see our Editorial Policies and the Editorial Policy Checklist.

Statistics

For all statistical analyses, confirm that the following items are present in the figure legend, table legend, main text, or Methods section.

n/a	Confirmed	
☐	☒	The exact sample size (*n*) for each experimental group/condition, given as a discrete number and unit of measurement
☐	☒	A statement on whether measurements were taken from distinct samples or whether the same sample was measured repeatedly
☐	☒	The statistical test(s) used AND whether they are one- or two-sided<br>*Only common tests should be described solely by name; describe more complex techniques in the Methods section.*
☒	☐	A description of all covariates tested
☐	☒	A description of any assumptions or corrections, such as tests of normality and adjustment for multiple comparisons
☐	☒	A full description of the statistical parameters including central tendency (e.g. means) or other basic estimates (e.g. regression coefficient) AND variation (e.g. standard deviation) or associated estimates of uncertainty (e.g. confidence intervals)
☐	☒	For null hypothesis testing, the test statistic (e.g. *F*, *t*, *r*) with confidence intervals, effect sizes, degrees of freedom and *P* value noted<br>*Give P values as exact values whenever suitable.*
☒	☐	For Bayesian analysis, information on the choice of priors and Markov chain Monte Carlo settings
☒	☐	For hierarchical and complex designs, identification of the appropriate level for tests and full reporting of outcomes
☒	☐	Estimates of effect sizes (e.g. Cohen's *d*, Pearson's *r*), indicating how they were calculated

Our web collection on statistics for biologists contains articles on many of the points above.

Software and code

Policy information about availability of computer code

Data collection	NIS-Elements 5.11.03
Data analysis	ImageJ 1.53c, GraphPad PRISM 9, STAR 2.6.1d, DESeq2_1.32.0, GSEA v4.1.0, R v4.1, fgsea v1.18.0

For manuscripts utilizing custom algorithms or software that are central to the research but not yet described in published literature, software must be made available to editors and reviewers. We strongly encourage code deposition in a community repository (e.g. GitHub). See the Nature Portfolio guidelines for submitting code & software for further information.

Data

Policy information about availability of data

All manuscripts must include a data availability statement. This statement should provide the following information, where applicable:

- Accession codes, unique identifiers, or web links for publicly available datasets
- A description of any restrictions on data availability
- For clinical datasets or third party data, please ensure that the statement adheres to our policy

The raw transcriptome data generated in this study RNA-Seq data have been deposited with the NCBI's Gene Expression Omnibus under accession code (#GSE196535)[https://www.ncbi.nlm.nih.gov/geo/query/acc.cgi?acc=GSE196535]. Overexpression plasmids generated in this study were deposited with the Addgene depository (IDs 190133 and 190134). Summary statistics for GWAS were downloaded from https://www.ebi.ac.uk/gwas/ with accession codes GCST006900 [https://www.ebi.ac.uk/gwas/studies/GCST006900], GCST008996 [https://www.ebi.ac.uk/gwas/studies/GCST008996], GCST90002232 [https://www.ebi.ac.uk/gwas/studies/GCST90002232], GCST90002238 [https://www.ebi.ac.uk/gwas/studies/GCST90002238], from http://diagram-consortium.org/ (data described as "T2D GWAS meta-analysis – Genetic Credible Sets"), and dbGaP with accession number phs001672.v1.p1 [https://www.ncbi.nlm.nih.gov/projects/gap/cgi-bin/study.cgi?study_id=phs001672.v1.p1]. All data supporting the findings of this study are available within the article and Supplementary Information files. Any

other data or materials generated or used in this study are available from the corresponding authors upon reasonable request. Source data are provided with this paper.

Field-specific reporting

Please select the one below that is the best fit for your research. If you are not sure, read the appropriate sections before making your selection.

[x] Life sciences [ ] Behavioural & social sciences [ ] Ecological, evolutionary & environmental sciences

For a reference copy of the document with all sections, see nature.com/documents/nr-reporting-summary-flat.pdf

Life sciences study design

All studies must disclose on these points even when the disclosure is negative.

Sample size	The sample sizes for animal studies were determined using power estimation using GPower v3.1. At least a minimum of 3 biological replicates were used.
Data exclusions	Predetermined exclusion criteria of samples identified using ROUT method with Q=1% was applied across analyses, and resulted in the exclusion of <3% of samples.
Replication	The animal experiments comparing WT and KO animals were repeated at least three times to collect sufficient material and datapoints for different analyses and confirm the results which were consistent across repeated experiments. The overexpression in vivo was conducted in at least 7 animals per group. Experiments involving cells were performed three times independently and provided consistent data.
Randomization	Before the start of animal experiments, littermates of the same genotype were randomly assigned to different diets. For the overexpression in vivo study, knockout mice were randomly assigned to the two groups. For experiments not involving animals, allocation to experimental groups was randomized.
Blinding	Histological quantifications and quantification of fluorescence were conducted in a blinded manner. All analyses on the overexpression in vivo were blinded, AAV8 variant used was blinded to the investigators during sample collection and analysis. The analyses of mice fed standard vs. high-fat diet could not be blinded as the mice were separated in different cages by diet. However, the genotype was blinded to the researchers. For experiments not involving animals, the investigators were blinded to group allocation during data collection and analysis.

Reporting for specific materials, systems and methods

We require information from authors about some types of materials, experimental systems and methods used in many studies. Here, indicate whether each material, system or method listed is relevant to your study. If you are not sure if a list item applies to your research, read the appropriate section before selecting a response.

Materials & experimental systems

n/a	Involved in the study	
[ ]	[x]	Antibodies
[ ]	[x]	Eukaryotic cell lines
[x]	[ ]	Palaeontology and archaeology
[ ]	[x]	Animals and other organisms
[x]	[ ]	Human research participants
[x]	[ ]	Clinical data
[x]	[ ]	Dual use research of concern

Methods

n/a	Involved in the study	
[x]	[ ]	ChIP-seq
[x]	[ ]	Flow cytometry
[x]	[ ]	MRI-based neuroimaging

Antibodies

Antibodies used

The following antibodies were used:
anti-HA antibody conjugated to AlexaFluor647 (BioLegend, clone 16B12, #682404), 1:1,000 (IF)
anti-CREB (48H2)(Cell Signaling, #9197), 1:1,000 (WB)
anti-phospho-CREB (Millipore Sigma, #06-519), 1:1,000 (WB)
anti-Akt (E7J2C)(Cell Signaling, #58295), 1:1,000 (WB)
anti-phospho-Akt (D9E)(Cell Signaling, #4060), 1:1,000 (WB)
anti-PEPCK (Abcam, ab70358), 1:1,000 (WB)
anti-S6 (54D2)(Cell Signaling, #2317), 1:1,000 (WB)
anti-phospho-S6 (Cell Signaling, #2211), 1:1,000 (WB)
anti-β-actin (C-4)(Santa Cruz, sc-47778), 1:15,000 (WB)
anti-rabbit IRDye 680RD (#925-68071, Li-cor), 1:10,000 (WB)
anti-mouse IRDye 800CW (#925-32210, Li-cor), 1:10,000 (WB)
anti-mouse HRP-conjugated (Cell Signaling, #7076), 1:10,000 (WB)

	anti-rabbit HRP-conjugated (Cell Signaling, #7074) 1:10,000 (WB)
Validation	All antibodies were commercially obtained, and validation was based on the datasheets from the manufacturer as well as the extensive usage in the field. Specifically:<br>BioLegend, #682404, https://www.biolegend.com/ja-jp/products/alexa-fluor-647-anti-ha-11-epitope-tag-antibody-12506<br>Cell Signaling, #9197, https://www.cellsignal.com/products/primary-antibodies/creb-48h2-rabbit-mab/9197<br>Millipore Sigma, #06-519, https://www.emdmillipore.com/US/en/product/Anti-phospho-CREB-Ser133-Antibody,MM_NF-06-519<br>Cell Signaling, #58295, https://www.cellsignal.com/products/primary-antibodies/akt-pan-e7j2c-mouse-mab/58295<br>Cell Signaling, #4060, https://www.cellsignal.com/products/primary-antibodies/phospho-akt-ser473-d9e-xp-rabbit-mab/4060<br>Abcam, ab70358, https://www.abcam.com/pck1pepc-antibody-ab70358.html<br>Cell Signaling, #2317, https://www.cellsignal.com/products/primary-antibodies/s6-ribosomal-protein-54d2-mouse-mab/2317<br>Cell Signaling, #2211, https://www.cellsignal.com/products/primary-antibodies/phospho-s6-ribosomal-protein-ser235-236-antibody/2211<br>Santa Cruz, sc-47778, https://www.scbt.com/p/beta-actin-antibody-c4<br>#925-68071, Li-cor, https://www.licor.com/bio/reagents/irdye-680rd-goat-anti-rabbit-igg-secondary-antibody<br>#925-32210, Li-cor, https://www.licor.com/bio/reagents/irdye-800cw-goat-anti-mouse-igg-secondary-antibody<br>Cell Signaling, #7076, https://www.cellsignal.com/products/secondary-antibodies/anti-mouse-igg-hrp-linked-antibody/7076<br>Cell Signaling, #7074, https://www.cellsignal.com/products/secondary-antibodies/anti-rabbit-igg-hrp-linked-antibody/7074

Eukaryotic cell lines

Policy information about cell lines

Cell line source(s)	HEK293T: ATCC CRL-1573.
Authentication	Not authenticated independently of ATCC. ATCC authentication by STR profiling.
Mycoplasma contamination	cell line tested negative for Mycoplasma using a PCR test
Commonly misidentified lines (See ICLAC register)	No commonly misidentified cell lines were used.

Animals and other organisms

Policy information about studies involving animals; ARRIVE guidelines recommended for reporting animal research

Laboratory animals	Wild-type C57BL/6J mice and Gpr151 knock-out line (on C57BL/6J background) were used. Both males and females were used, between 8 and 17 weeks old.
Wild animals	The study did not involve wild animals.
Field-collected samples	The study did not involve samples collected in the field.
Ethics oversight	All animal studies were approved by the Administrative Panel on Laboratory Animal Care at Stanford University, and were performed according to the guidelines of the American Association for the Accreditation of Laboratory Animal Care.

Note that full information on the approval of the study protocol must also be provided in the manuscript.
